# Identification of promising chickpea interspecific derivatives for agro-morphological and major biotic traits

**DOI:** 10.3389/fpls.2022.941372

**Published:** 2022-08-04

**Authors:** Mohar Singh, Tapan Kumar, Salej Sood, Nikhil Malhotra, Upasana Rani, Sarvjeet Singh, Inderjit Singh, Shayla Bindra, Sanjeev Kumar, Sandeep Kumar

**Affiliations:** ^1^Indian Council of Agricultural Research (ICAR)-National Bureau of Plant Genetic Resources Regional Station, Shimla, India; ^2^International Centre for Agricultural Research in Dry Areas (ICARDA)-Food Legume Research Platform, Bhopal, India; ^3^Indian Council of Agricultural Research (ICAR)-Central Potato Research Institute, Shimla, India; ^4^Department of Plant Breeding and Genetics, Punjab Agricultural University, Ludhiana, India; ^5^Department of Plant Breeding and Genetics, Sher-e-Kashmir University of Agricultural Sciences and Technology, Jammu, India; ^6^Indian Council of Agricultural Research (ICAR)-National Bureau of Plant Genetic Resources, New Delhi, India

**Keywords:** transgressive segregation, fruitful heterosis, wild *Cicer*, genetic improvement, biotic stress

## Abstract

The wild *Cicer* species is well-known for having climate-resilient and productivity-enhancing traits of interest. Therefore, wide hybridization could be used as a realistic strategy for introgressing prospective traits from wild species into the cultivated gene pool. The present study was, thus, undertaken to evaluate F_7_ chickpea interspecific derivatives derived from *Cicer reticulatum* Ladiz. and *C. echinospermum* P. H. Davis wild annual *Cicer* species. As a result, a set of six interspecific crosses were advanced using the single seed descent (SSD) method of breeding. The F_7_ generation of these crosses was assessed in two diverse agro-ecological regions of India. The data revealed a wide range of variation with respect to seed yield and its important component traits, which resulted in the identification of the most promising derivatives carrying desirable characters as indicated by range, mean, and coefficient of variation. Further, fruitful heterosis was also estimated as promising selection criteria for identifying superior lines for earliness and high seed yield, including resistance against prevailing stresses (ascochyta blight, botrytis gray mold, dry root rot, and fusarium wilt). The superior derivatives carrying putative characters could be recommended for further breeding and selection of genetic materials for developing suitable genotypes.

## Introduction

Chickpea (*Cicer arietinum* L.) is a true diploid (2n = 2x = 16) annual food legume species having a genome size of ~738 Mbp (Varshney et al., 2012). The domesticated species has evolved from its immediate wild progenitor *C. reticulatum* Ladiz. through the natural selection process. The genus *Cicer* consists of 9 annual and 35 perennial species (van der Maesen, 1987). Recently, Toker et al. (2021) introduced one more wild annual species *C. turcicum* Toker, Berger and Gokturk, thereby increasing the count to 10 annual species in the existing gene pool. It is a winter-season legume crop that thrives at temperatures between 20 and 25°C during the day and 15–20°C at night temperatures, thereby performing well in drier weather conditions (Saraf et al., 1998). In the last two decades (1995–2016), the global chickpea area has expanded by 8%, grain yield by 27%, and overall production by 40% (Food and Agriculture Organization, 2020). India, being the world's largest producer of chickpea, accounts for ~70% of total annual pulse production of ~12 million tons, which was harvested from ~11 million ha, which is far less than its true potential when compared to other major crops (Dixit, 2018). The main causes of such trivial gains are a variety of stresses that limit yield and stability (Siddique et al., 2000; Varshney et al., 2012). Furthermore, the non-availability of genetically improved crop varieties remains a serious concern in achieving desired production levels even in highly productive environments (Chaturvedi and Nadarajan, 2010). A narrow gene pool, due to its single domestication event and a high rate of self-pollination, is another constraint in breeding cultivated chickpea (Abbo et al., 2003, 2005). However, resistant sources to major biotic and abiotic stresses, including yield-related traits, are often not available within cultivated germplasm due to the domestication bottleneck, which has bifurcated the interest of chickpea researchers to use crop wild relatives (CWRs) for its genetic improvement (Croser et al., 2003; Mallikarjuna et al., 2007; Bakir et al., 2021; Vance et al., 2021; Sari et al., 2022). In the past, wild progenitors have been introgressed as potential donors of productivity-enhancement related characters in some other crop species, such as rice (Xiao et al., 1996) and tomato (Tanksley and McCouch, 1996). Therefore, to attain further breakthroughs for improving yield and stability in future crop varieties, new traits of interest must be incorporated into the cultivated background of chickpea. Moreover, it is also imperative to accumulate other complimentary genes and alleles from wild relatives to diversify the cultivated gene pool and maximize genetic gains from selection (Vega and Frey, 1980). The wild *Cicer* species consists of target characters for distinct morphological features and resistance against biotic and abiotic stresses (Robertson et al., 1997; Singh and Ocampo, 1997; Singh et al., 2005, 2014, 2021; Sandhu et al., 2006; Talip et al., 2018; Toker et al., 2021). As far as their crossability with cultivated chickpea is concerned, several cross-combination studies involving *C. reticulatum* and *C. echinospermum* have exhibited higher variability for important yield-related and stress-resistant traits (Koseoglu et al., 2017; Singh et al., 2018). So, an immediate thrust is required to broaden the genetic base of domesticated chickpea cultivars using the introgression of wild *Cicer* species. Consequently, the multi-location evaluation of interspecific derivatives (IDs) will aid in the identification of promising and stable recombinants across environments (Rakshit et al., 2012). It would also help in the identification of an optimal environment through which the stability of improved progenies could be assessed. Hence, the present study was carried out to evaluate and identify potential interspecific derivatives of chickpea for yield and its important component traits, including major biotic stress factors under two agro-ecological regions of India.

## Materials and methods

### Genetic materials, population development, and testing

The genetic materials included in the present study were three cultivated chickpea varieties Pusa372, PBG5, and BGD72 of *C. arietinum*, selected as recipients with two wild annual *Cicer* species, ILWC229 (EC720438) of *C. reticulatum* and ILWC246 (EC720481) of *C. echinospermum*, which were chosen as donor parents for interspecific hybridization. These wild annual *Cicer* species were selected on the basis of their resistance against ascochyta blight [*Ascochyta rabiei* (Pass.) Labr.], and botrytis gray mold (*Botrytis cinerea* Pers. ex. Fr.), including a high number of seeds plant^−1^ (Singh et al., 2014, 2018). These wild accessions have also been tested for their resistance against fusarium wilt [*Fusarium oxysporum* f. sp. *ciceris* (Padwick) Matuo and K. Sato] and dry root rot [*Rhizoctonia bataticola* (Taub.) E.J. Butler] (unpublished results). The wide hybridization experiments were undertaken at Indian Council of Agricultural Research (ICAR)-National Bureau of Plant Genetic Resources, Pusa New Delhi (28° 35′ N′, 70° 18′ E, 226 m amsl) and the Mountain Agricultural Research and Extension Center (MAREC) of Chaudhary Sarwan Kumar, Himachal Pradesh Krishi Vishvavidyalaya, Sangla (31° 55′ and 32° 20′ N and 77° 00′ and 79° 50′ E, 2758 m amsl), Himachal Pradesh, India during the winter season of 2012-13 and summer 2013. As a result, a total of six interspecific cross-combinations of Pusa372 × EC720438, PBG5 × EC720438, BGD72 × EC720438, BGD72 × EC720481, PBG5 × EC720481, and Pusa372 × EC720481 were successfully obtained as F_1_ hybrid seeds. Subsequently, the hybridity of true-to-type interspecific crosses were confirmed using distinct morphological and molecular markers (unpublished results). From F_2_ onward, value-added progenies of these interspecific derivatives (IDs) were advanced upto the F_7_ stage through the single seed descent (SSD) method of breeding (Goulden, 1939). Further, during the winter season of 2019–2020, the F_7_ IDs of Pusa372 × EC720438, PBG5 × EC720438, BGD72 × EC720438, BGD72 × EC720481, PBG5 × EC720481, and Pusa372 × EC720481 were planted in an experimental research farm of two different agro-ecological locations viz. International Center for Agricultural Research in Dry Areas (ICARDA) Pulse Research Platform, Bhopal (23° 10′N, 76° 88'E, 498 m amsl) and Punjab Agricultural University (PAU), Ludhiana (30° 54′N, 75° 48′E, 247 m amsl) of India. The agro-climatic description of each location is also given in Table 1. The experiments were undertaken in Augmented Block Design (Federer, 1956), and the performance was compared with regional and local checks of respective locations (susceptible JG62 and resistant PBG7 checks at Ludhiana, India, and resistant checks JG14, BG3043, and RVG202 at Bhopal, India). Seeds of each ID were sown in 3-m long rows spaced at 10 cm (plant to plant) and 40 cm (row to row) apart. One pre-sowing irrigation was also given to ensure satisfactory seed germination. Furthermore, recommended agronomic practices were followed for raising the chickpea genetic materials. During the whole cropping season, 2 natural rains were also experienced and hence the necessity of manual irrigation was not felt. The observations were recorded on five competitive plants from each regional and local check and 15 plants from each F_7_ IDs of all six crosses. The agro-morphological data were taken on days to 50% flowering (DF), days to 80% maturity (DM), plant height (cm) (PH), number of branches plant^−1^ (NBPP), number of seeds pod^−1^ (NSPP), 100-seed weight (g) (SW), seed yield plant^−1^ (g) (SYPP), and biological yield plant^−1^ (g) (BYPP). Fruitful heterosis was also estimated following Koseoglu et al. (2017) as H_F_ (%) = [(F_7_-BP)/BP] × 100, where, F_7_ is 7th generation of IDs and BP is the mean of the better parent.

**Table 1:** Agro-climatic description of the Indian locations where chickpea interspecific derivatives were evaluated during the study period of winter 2019–2020 and 2020–2021.

**Location**	**Latitude**	**Longitude**	**Elevation (m asl)**	**Mean rainfall (mm)**	**Temperature** **°C**		**Soil type**
					**Min**	**Max**	
Bhopal	23° 10′N	76° 88′E	498	9.67	19.33	29.83	Medium dark black
Ludhiana	30° 54′N	75° 48′E	247	55.7	11.00	24.7	Sandy loam

m asl, meters above sea level.

### Screening against major biotic stresses

#### Ascochyta blight (*A. rabiei*)

Disease reaction of chickpea interspecific derivatives to ascochyta blight was organized at the Experimental Farm of Punjab Agricultural University, Ludhiana, India (30° 54'N, 75° 48′E, 247 m amsl) during the winter season of 2019–2020. All derivatives belonging to six interspecific crosses were planted in 3-m long rows spaced at 10 cm (plant to plant) and 40 cm (row to row) apart. All plant populations were artificially inoculated by frequently spraying with ascosporic suspension (1 × 10^6^ spores ml^−1^) using local isolate of *A. rabiei*. The observations were recorded on terminal disease reaction at the vegetative and reproductive plant stages on a rating scale of 1–9, as suggested by Pande et al. (2010). Based on disease screening, the interspecific derivatives were categorized for their reaction to pathogen infection as: 1 = asymptomatic (Free), 3 = resistant (R), 5 = moderately resistant (MR), 7 = susceptible (S), and 9 = highly susceptible (HS).

#### Botrytis gray mold (*B. cinerea*)

Botrytis gray mold (BGM) is a devastating pathogen of chickpea, especially in those regions where warm, cloudy, and humid weather situation persists. Several epidemics of BGM causing whole crop loss in chickpea growing in the areas of northwestern India have been reported (Singh et al., 1991). All the interspecific derivatives were screened at Ludhiana, India using cut-twig screening technique, in which water was used as supportive media. Three to four twigs of a single line from each derivative were taken and placed in a test tube having fresh tap water. Subsequently, twigs were inoculated by spraying spore suspension of *B. cinerea* (10,000 spores ml^−1^) and covered with moist chambers for 144 h. An incubation period of 8 h dark and 16 h light was provided with fluorescent lamps [60 by 3.75 cm (24 × 1.5 in.); 20 W, 32 lumens W^−1^]. Further, observations on disease incidence against BGM were assessed using a 1–9 rating scale (Singh et al., 1991) after 6 days of inoculation where: 1 = asymptomatic (Free), 3 = resistant (R), 5 = moderately resistant (MR), 7 = susceptible (S), and 9 = highly susceptible (HS).

#### Dry root rot [*R. bataticola* (Taub.)]

Dry root rot is more dominant when the crop is exposed to drought stress (Rai et al., 2022). The symptoms of this pathogen are obvious during the post-flowering stage, leading to drooping and chlorosis of petioles and leaves as well. The leaves and stems of affected plants are generally straw-colored, and in some cases, the lower leaves and stems show brown discoloration and the tap root becomes dark. For screening of chickpea interspecific derivatives at Bhopal, India (23° 10′N, 76° 88′E, 498 m amsl), inoculated seedlings were observed and the data on disease severity was recorded using a 1–9 rating scale developed by Nene et al. (1991), where 1 = asymptomatic (Free), 3 = resistant (R), 5 = moderately resistant (MR), 7 = susceptible (S), and 9 = highly susceptible (HS).

#### Fusarium wilt (*F. oxysporum*)

Fusarium wilt is another devastating disease of chickpea, and the selection of highly resistant interspecific derivatives among enhanced progenies is the prime concern of this study. All F_7_ derivatives belonging to six wide crosses were screened under real field conditions at Bhopal, India. However, resistant and susceptible checks were also repeated after 20 rows of each replication. Observations on disease incidence were recorded using 1–9 rating scale (Nene and Haware, 1980) as: 1 = asymptomic (Free); 3 = resistant (R); 5 = moderately resistant (MR); 7 = susceptible (S); 9 = highly susceptible (HS). Wilt incidence percentage was also observed during the flowering and pod filling stages, as described by Biswas and Jubayer Ali (2017).

### Statistical analyses

ANOVA for augmented design was carried out using R package “augmented RCBD” (Aravind et al., 2020). The linear mixed models were implemented in lmer from package lme4 of R using REML to calculate BLUEs and BLUPs and estimates of the variance components (R Core Team, 2018). The adjusted means of all the quantitative traits were used for the estimation of principal component analysis, cluster analysis, and correlations. PCA and correlation studies were done using ggplot and cor function in R, respectively. The R package “corrplot” was used for the depiction of correlation plots. The phenotypic and genotypic coefficients of variation (PCV and GCV) for different traits were calculated as PCV = √VP/ mean × 100, GCV = √VG/mean × 100 as per Burton (1952). Heritability (bs) was estimated as h^2^ (ns) = √A/VP × 100 as per Lush (1940). The expected genetic advance was calculated as EGA = k × VG/VP × √VP, as per the procedure of Johnson et al. (1955), where k = 2.06 (standard value assumed at 5% selection intensity), VG is the genotypic variance, and VP is the phenotypic variance. However, fruitful heterosis (H_F_) coined by Koseoglu et al. (2017) was also estimated over better parent (BP) for identifying superior derivatives in F_7_ generation as: H_F_ (%) = [(F_7_-BP)/BP] × 100%, where, BP is the mean value of the better parent of a particular cross.

## Results

### Agro-morphological evaluation

**Figure 1:**
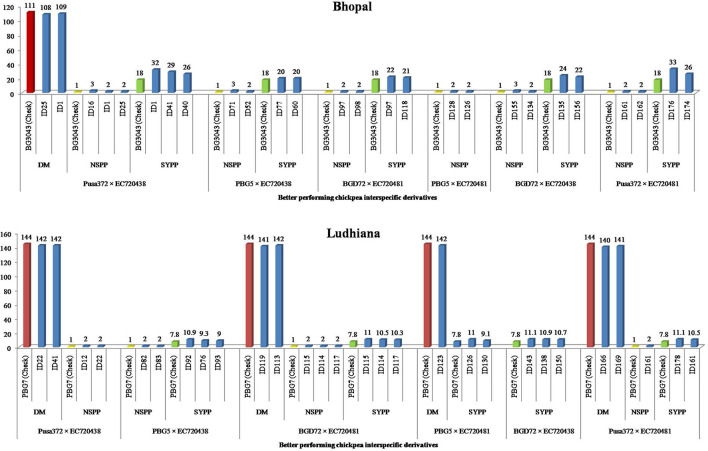
Performance of chickpea interspecific derivatives among different crosses and checks for major agro-morphological traits under two agro-ecological conditions of India. DM, days to maturity; NSPP, number of seeds pod^−1^; SYPP, seed yield plant^−1^.

Linear mixed model analysis showed significant differences among genotypes for all the traits, except days to maturity and number of seeds pod^−1^. The combined analysis of variance indicated significant variation for block, treatment, and interaction among test entries for days to flowering, days to maturity, the number of branches plant^−1^, plant height, and seed yield plant^−1^ at Bhopal, India (Table 2, Supplementary Table 1). Likewise, it was also significant for all the characters in Ludhiana, India (Table 2; Supplementary Table 1). However, the summary of descriptive statistical parameters revealed a wide range of variation with respect to important agro-morphological characters as also manifested by the range of variation. The distribution of genetic materials was highly skewed and significant for all the characters at both locations (Table 3). Further, the F_7_ interspecific derivatives of cross Pusa372 × EC720438, PBG5 × EC720438, BGD72 × EC720438, BGD72 × EC720481, PBG5 × EC720481, and Pusa372 × EC720481 exhibited a wide range of variation, as also reflected by the range, mean, and coefficient of variation (Table 4). The results showed sufficient variability among enhanced progenies of interspecific derivatives developed from *C. reticulatum* and *C. echinospermum* species. In general, days to flowering and maturity exhibited variation in two locations as the genetic materials flowered and matured early in Bhopal, India than Ludhiana, India. The maximum average plant height was observed in the cross of BGD72 × EC720481 followed by PBG5 × EC720481, PBG5 × EC720438, and minimum in cross Pusa372 × EC720438 and Pusa372 × EC720481 at both the locations (Table 4). As far as the number of seeds pod^−1^ is concerned, it was reported as an average of one to two seeds pod^−1^. The trait 100-seed weight revealed substantial variation in different interspecific derivatives and maximum 100-seed weight was reported in the cross of BGD72 × EC720438 and minimum in Pusa372 × EC720438 at both the locations (Table 4). The seed yield plant^−1^, being a very important economic trait of interest, also manifested variation ranging from the cross PBG5 × EC720438 (15.30 g) to Pusa372 × EC720438 (18.91 g) in Bhopal, India. Likewise, cross PBG5 × EC720481 and BGD72 × EC720481 exhibited maximum seed yield plant^−1^ in Ludhiana, India. In general, the extent of genetic parameters indicated that the magnitude of phenotypic variances and coefficient of variation was higher than genotypic variances for the majority of the traits (Table 5). Likewise, heritability was higher for all the agro-morphological characters, but comparatively genetic advance along with gain as a percentage of mean was low in magnitude. The summary of identifying promising interspecific derivatives for important agro-morphological traits has been depicted in Figure 1.

**Table 2:** Analysis of variance of chickpea interspecific derivatives for seed yield and its important component traits at Bhopal and Ludhiana, India.

**Source**	**DF**	**DM**	**NBPP**	**PH**	**SYPP**
**Bhopal**					
Block (ignoring treatments)	548.48**	835.29**	66.49**	708.87**	59.02**
Treatment (eliminating blocks)	28.79**	21.22**	50.63**	40.42**	14.66**
Treatment: check	52.33**	14.33**	212.05**	169.9**	43.76**
Treatment: test and test vs. check	28.52**	21.3**	48.83**	38.97**	14.34**
**Ludhiana**					
Block (ignoring treatments)	164.1**	30.64**	21.03**	333.43**	100.84**
Treatment (eliminating blocks)	38.66**	8.44**	2.11*	25.48**	2.53**
Treatment: check	228**	16.33*	4.62*	202.87**	13.53**
Treatment: test and test vs. check	36.54**	8.35**	2.08*	23.5**	2.41**

* p = 0.01,

** p = 0.05, ns, non-significant.

**Table 3:** Summary of descriptive statistical parameters for important agro-morphological traits.

**Trait**	**Min**	**Max**	**Mean**	**Std Error**	**Std Deviation**	**Skewness**	**Kurtosis**
**Bhopal**							
DF	61.14	90.14	74.87	0.49	6.55	−0.36^*^	2.24^**^
DM	109.05	128.38	120.57	0.47	6.34	−0.4*	1.66**
NBPP	7.95	38.57	15.14	0.38	5.07	1.71**	7.13**
PH	31.29	68.62	54.46	0.6	8.14	−0.58**	2.59 ns
SYPP	8.95	36.29	16.07	0.32	4.29	1.09**	5.22**
**Ludhiana**							
DF	80	106.71	97.41	0.37	4.98	−0.6**	3.36 ns
DM	142.62	155.62	147.91	0.23	3.05	0.39*	2.37*
NBPP	6	16	11.07	0.12	1.67	0.37*	3.2 ns
PH	31.62	59.82	46.71	0.39	5.26	−0.17 ns	2.94 ns
SYPP	2.87	10.97	6.07	0.17	2.35	0.22 ns	1.91**

* p = 0.01,

** p = 0.05, ns, non-significant.

**Table 4:** Range, mean, standard error, and coefficient of variation of agro-morphological traits in F_**7**_ generation of chickpea interspecific derivatives at Bhopal and Ludhiana, India.

	**Bhopal**			**Ludhiana**		
**Trait/Cross**	**Range**	**Mean ±SE**	**CV%**	**Range**	**Mean ±SE**	**CV%**
**Days to flowering**						
Pusa372 × EC720438	61.00–74.00	67.11 ± 0.43	4.50	86.00–102.00	94.80 ± 0.50	3.70
PBG5 × EC720438	65.50–82.00	77.18 ± 0.52	4.58	96.00–104.00	100.93 ± 0.35	2.37
BGD72 × EC720481	72.50–84.00	78.95 ± 0.66	4.24	88.00–106.00	98.42 ± 0.82	4.26
PBG5 × EC720481	81.00–87.50	83.75 ± 0.71	2.39	93.00–103.00	99.25 ± 1.32	3.76
BGD72 × EC720438	72.00–88.00	76.68 ± 0.68	4.83	88.00–106.00	98.07 ± 0.89	4.97
Pusa372 × EC720481	67.00–80.50	74.47 ± 0.71	4.14	87.00–106.00	94.74 ± 1.40	6.43
**Days to maturity**						
Pusa372 × EC720438	108.50–116.50	112.92 ± 0.26	1.67	142.00–151.00	148.08 ± 0.39	1.85
PBG5 × EC720438	113.00–128.00	124.96 ± 0.45	2.42	144.00–154.00	148.98 ± 0.40	1.80
BGD72 × EC720481	118.00–128.00	126.12 ± 0.41	1.67	141.00–151.00	148.46 ± 0.49	1.70
PBG5 × EC720481	123.00–128.00	125.50 ± 0.50	1.13	142.00–151.00	146.00 ± 0.87	1.68
BGD72 × EC720438	115.00–128.00	122.00 ± 0.70	3.15	145.00–151.00	147.73 ± 0.54	2.02
Pusa372 × EC720481	116.00–126.00	120.58 ± 0.54	1.94	140.00–151.00	142.63 ± 0.58	1.75
**Plant height (cm)**						
Pusa372 × EC720438	33.00–62.00	46.12 ± 0.96	14.74	30.66–55.00	42.47 ± 0.76	12.60
PBG5 × EC720438	44.00–66.50	59.11 ± 0.61	6.98	38.33–57.33	49.17 ± 0.59	8.07
BGD72 × EC720481	50.50–66.50	60.65 ± 0.73	6.11	40.33–59.33	50.23 ± 0.85	8.67
PBG5 × EC720481	57.00–63.50	59.56 ± 0.68	3.22	47.00–56.33	50.17 ± 1.24	7.00
BGD72 × EC720438	44.50–70.50	56.43 ± 1.03	9.96	41.66–56.00	49.15 ± 0.63	7.04
Pusa372 × EC720481	43.50–62.50	52.81 ± 1.29	10.64	35.33–51.33	42.80 ± 0.89	9.10
**No. of branches plant** ^−1^						
Pusa372 × EC720438	10.50–17.00	13.97 ± 0.77	11.54	6.33–11.66	09.69 ± 0.15	11.25
PBG5 × EC720438	11.00–27.00	15.01 ± 0.48	21.82	8.66–15.00	11.43 ± 0.19	11.15
BGD72 × EC720481	11.50–19.50	15.38 ± 0.45	14.83	8.99–13.66	11.31 ± 0.25	11.26
PBG5 × EC720481	11.50–16.50	12.94 ± 0.55	11.95	7.99–11.66	10.58 ± 0.44	11.89
BGD72 × EC720438	11.00–26.50	16.43 ± 0.75	25.16	8.66–15.66	12.04 ± 0.35	15.87
Pusa372 × EC720481	11.50–19.50	15.68 ± 0.61	16.98	9.66–15.30	12.20 ± 0.41	14.67
**No. of seeds pod** ^−1^						
Pusa372 × EC720438	1.00–3.00	1.80 ± 0.06	25.10	1.00–1.90	1.30 ± 0.04	19.78
PBG5 × EC720438	1.00–3.00	1.93 ± 0.05	16.89	1.10–1.90	1.42 ± 0.03	16.24
BGD72 × EC720481	1.00–2.00	1.81 ± 0.08	22.23	1.00–1.90	1.50 ± 0.04	14.82
PBG5 × EC720481	2.00–2.00	2.00 ± 0.00	0.00	1.20–1.80	1.45 ± 0.06	12.23
BGD72 × EC720438	1.00–3.00	1.73 ± 0.10	30.05	1.00–1.80	1.35 ± 0.04	17.00
Pusa372 × EC720481	1.00–2.00	1.53 ± 0.12	33.61	1.00–1.90	1.47 ± 0.05	15.24
**100-seed weight (g)**						
Pusa372 × EC720438	09.42–20.48	13.59 ± 0.38	19.89	10.40–18.00	14.07 ± 0.19	9.35
PBG5 × EC720438	12.30–20.64	19.24 ± 0.18	6.29	13.20–28.60	17.38 ± 0.33	12.91
BGD72 × EC720481	17.76–21.84	19.47 ± 0.18	4.62	15.20–20.20	17.75 ± 0.23	6.63
PBG5 × EC720481	18.08–20.68	19.60 ± 0.28	4.03	17.30–19.50	18.61 ± 0.25	3.86
BGD72 × EC720438	17.48–23.44	21.21 ± 0.23	6.06	16.30–31.50	23.58 ± 0.88	20.42
Pusa372 × EC720481	13.28–20.38	16.63 ± 0.52	13.75	11.90–19.30	15.87 ± 0.49	13.45
**Seed yield plant**^−1^ **(g)**						
Pusa372 × EC720438	10.63–31.83	18.91 ± 0.49	18.15	3.00–6.43	3.36 ± 0.07	15.47
PBG5 × EC720438	12.23–19.50	15.30 ± 0.28	12.46	3.13–10.92	6.53 ± 0.26	27.44
BGD72 × EC720481	13.00–21.50	15.45 ± 0.46	15.06	0.02–11.00	7.60 ± 0.51	34.15
PBG5 × EC720481	13.68–18.50	16.13 ± 0.59	10.32	4.08–11.00	7.66 ±0.80	29.44
BGD72 × EC720438	10.70–23.50	16.74 ± 0.50	16.51	0.01–11.13	7.40 ± 0.51	38.04
Pusa372 × EC720481	11.00–32.50	16.37 ± 1.03	24.41	0.01–11.13	5.00 ± 0.62	53.68

**Table 5:** Mean, variance, phenotypic, genotypic, and environmental coefficient of variance, heritability, genetic advance, and genetic advance as percent of mean for important traits.

**Trait**	**Mean**	**GV**	**PV**	**EV**	**GCV**	**PCV**	**ECV**	**H (bs)**	**GA**	**GAM**
**Bhopal**										
DF	74.87	27.34	33.67	06.33	06.98	07.75	03.36	81.19	09.72	12.98
DM	120.57	39.01	39.67	00.67	05.18	05.22	00.68	98.32	12.78	10.60
NBPP	15.14	09.44	17.93	08.49	20.3	27.98	19.25	52.64	4.6	30.38
PH	54.46	54.26	56.72	02.46	13.53	13.83	02.88	95.66	14.86	27.29
SYPP	16.07	09.49	12.76	03.26	19.17	22.22	11.24	74.43	05.48	34.12
**Ludhiana**										
DF	97.41	20.86	21.69	00.83	04.69	04.78	00.94	96.16	09.24	09.48
DM	147.91	05.8	08.19	02.39	01.63	01.93	01.04	70.83	04.18	02.83
NBPP	11.07	02.05	02.78	00.73	12.93	15.06	07.72	73.74	02.54	22.91
PH	46.71	23.2	30.05	06.85	10.31	11.74	05.60	77.21	08.73	18.69
SYPP	06.07	05.68	05.8	00.12	39.25	39.67	05.76	97.89	04.86	80.11

PV, phenotypic variance; GV, genotypic variance; EV, extreme variability; GCV, genotypic coefficient of variation; PCV, phenotypic coefficient variation; ECV, extreme climate variability; H(bs), heritability in broad sense; GA, genetic advance; GAM, genetic advance over mean.

### Estimation of fruitful heterosis (%)

The nature and magnitude of fruitful heterosis (%) of F_7_ interspecific derivatives were also estimated for days to maturity, plant height, number of seeds pod^−1^, and seed yield plant^−1^ at both locations (Table 6). The extent of fruitful heterosis was calculated using the percentage of deviation of interspecific derivatives from a better parent (BP). At Bhopal, India, fruitful heterosis means performance ranged from −0.35% (Pusa372 × EC720438) to 12.33% (PBG5 × EC720481) for days to maturity. Likewise, it ranged from 0.31% (BGD72 × EC720481) to 2.04% (PBG5 × EC720438) for the same traits in Ludhiana, India. However, an average mean performance for plant height varied from −14.20% (Pusa372 × EC720438) to 16.32% (BGD72 × EC720481) in Bhopal, India, and it ranged from −6.95% (Pusa372 × EC720481) to 22.62% (PBG5 × EC720438) in Ludhiana, India. The estimation of fruitful heterosis for number of seeds pod^−1^ varied from 52.63% (Pusa372 × EC720481) to 100.00% (PBG5 × EC720481) in Bhopal, India and it varied from −35.56% (BGD72 × EC720438) to −11.99% (BGD72 × EC720481) in Ludhiana, India. There was a substantial variation for seed yield plant^−1^ that ranged from −46.75% (Pusa372 × EC720481) to −8.89% (Pusa372 × EC720438) in Bhopal, India, and it varied −55.50% (Pusa372 × EC720438) to 17.17% (BGD72 × EC720438) in Ludhiana, India.

**Table 6:** Estimates of fruitful heterosis (%) in F_7_ wide cross populations for agro-morphological traits.

	**Bhopal**			**Ludhiana**		
**Trait/Cross**	**Range**	**Mean ±SE**	**CV%**	**Range**	**Mean ±SE**	**CV%**
**Days to maturity**						
Pusa 372 × EC720438	(−3.56)−3.56	(−0.35) ± 0.25	(−500.39)	(−3.36)−6.16	0.40 ± 0.30	529.88
PBG5 × EC720438	0.89–14.29	11.64 ± 0.40	0.40	(−1.37)−5.48	2.04 ± 0.27	89.95
BGD72 × EC720481	4.89–13.78	12.24 ± 0.36	15.10	(−2.70)−3.38	0.31 ± 0.33	545.49
PBG5 × EC720481	9.82–14.29	12.33 ± 0.47	10.76	(−0.69)−4.14	0.69 ± 0.60	244.95
BGD72 × EC720438	3.13–14.29	8.96 ± 0.64	39.24	(−0.69)−6.90	1.89 ± 0.38	109.10
Pusa372 × EC720481	3.11–12.00	7.04 ± 0.52	31.93	(−0.69)−6.90	0.44 ± 0.40	402.67
**Plant height (cm)**						
Pusa 372 × EC720438	(−38.89)−14.81	(−14.20) ± 1.78	(−88.8)	(−32.85)−20.46	(−2.25) ± 1.67	(−524.89)
PBG5 × EC720438	(−13.73)−28.43	15.92 ± 1.19	50.83	(−4.41)−42.97	22.62 ± 1.46	43.77
BGD72 × EC720481	(−3.81)−43.33	16.32 ± 1.73	54.08	(−19.98)−17.72	(−0.34) ± 1.69	(−2,388.38)
PB5 × EC720481	8.57–20.95	13.45 ± 1.29	27.14	(−3.49)−15.67	3.01 ± 2.55	239.56
BGD72 × EC720438	(−16.82)−31.78	5.48 ± 1.92	191.63	(−9.43)−21.74	6.86 ± 1.37	109.77
Pusa372 × EC720481	(−21.62)−12.61	(−4.84) ± 2.32	(−209.39)	(−23.20)−11.59	(−6.95) ± 1.94	(−121.85)
**No. of seeds pod** ^−1^						
Pusa 372 × EC720438	0.00–200.00	82.00 ± 6.82	58.77	(−50.00)−5.56	(−31.53) ± 2.02	(−43.21)
PBG5 × EC720438	0.00–200.00	93.48 ± 4.82	34.95	(−35.29)−11.76	(−16.52) ± 2.00	(−82.12)
BGD72 × EC720481	0.00–100.00	80.77 ± 7.88	49.76	(−41.18)−11.76	(−11.99) ± 2.56	(−108.80)
PBG5 × EC720481	100.00–100.00	100.00 ± 0.00	0.00	(−36.84)–(−5.26)	(−23.68) ± 3.30	(−39.40)
BGD72 × EC720438	0.00–200.00	73.33 ± 9.51	71.02	(−52.38)–(−14.29)	(−35.56) ± 2.00	(−30.81)
Pusa372 × EC720481	0.00–100.00	52.63 ± 11.77	97.47	(−52.38)–(−9.52)	(−30.08) ± 2.44	(−35.43)
**Seed yield plant**^−1^ **(g)**						
Pusa 372 × EC720438	(−53.50)−39.28	(−8.89) ± 2.56	(−203.43)	(−61.47)–(−12.36)	(−55.50) ± 1.05	(−13.37)
PBG5 × EC720438	(−45.65)–(−3.91)	(−32.56) ± 1.35	(−28.03)	(−55.41)−55.41	(−7.09) ± 3.76	(−359.73)
BGD72 × EC720481	(−46.94)–(−12.24)	(−36.88) ± 1.90	(−26.25)	(−42.80)−54.21	9.58 ± 5.12	272.56
PBG5 × EC720481	(−44.18)–(−24.49)	(−34.18) ± 2.40	(−19.86)	(−25.86)−30.72	(−3.29) ± 7.31	(−629.07)
BGD72 × EC720438	(−53.48)−2.17	(−27.20) ± 2.19	(−44.18)	(−27.34)−53.21	17.17 ± 4.28	136.60
Pusa372 × EC720481	(−64.23)–(5.69)	(−46.75) ± 3.35	(−31.22)	(−52.20)−18.07	(−25.54) ± 4.26	(−72.72)

(–) negative range performance recorded in parentheses.

### Adjusted mean box plots

**Figure 2:**
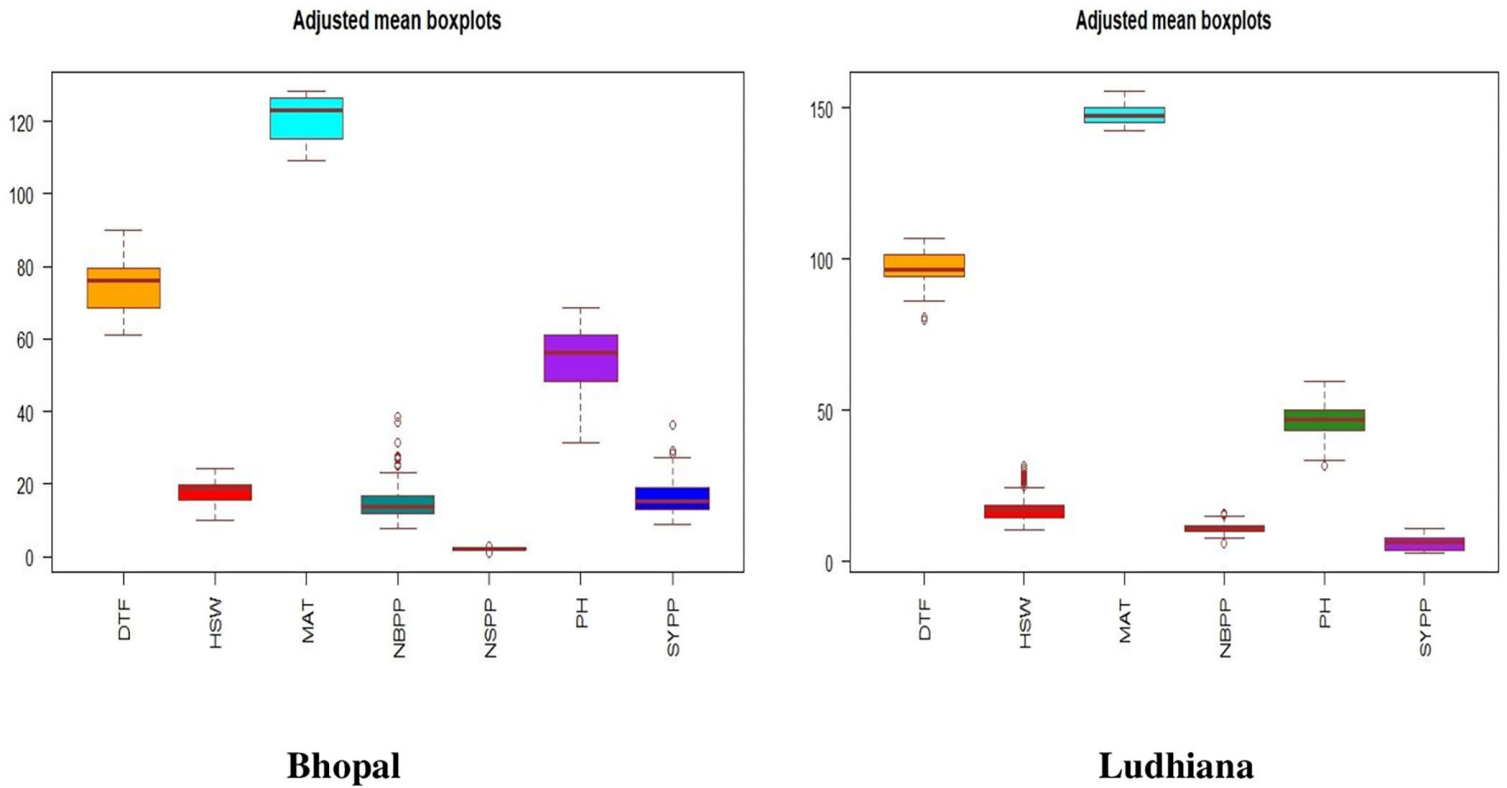
Adjusted mean boxplots of different agro-morphological traits for chickpea interspecific derivatives at Bhopal and Ludhiana, India. DTF, days to flowering; HSW, 100-seed weight; MAT, days to maturity, NBPP, number of branches plant^−1^; NSPP, number of seeds pod^−1^; PH, plant height; SYPP, seed yield plant^−1^.

The results on adjusted mean box plot performance exhibited that the chickpea interspecific derivatives exhibited a high degree of variation for days to flowering, days to maturity, and plant height, while the number of branches plant^−1^, 100-seed weight, and seed yield plant^−1^ showed lesser variations in Bhopal, India. The trend was similar in Ludhiana, India but the range of variation in data was tight, i.e., the variation in data was low in comparison to Bhopal, India (Figure 2).

### Correlations and principal component analysis

**Figure 3:**
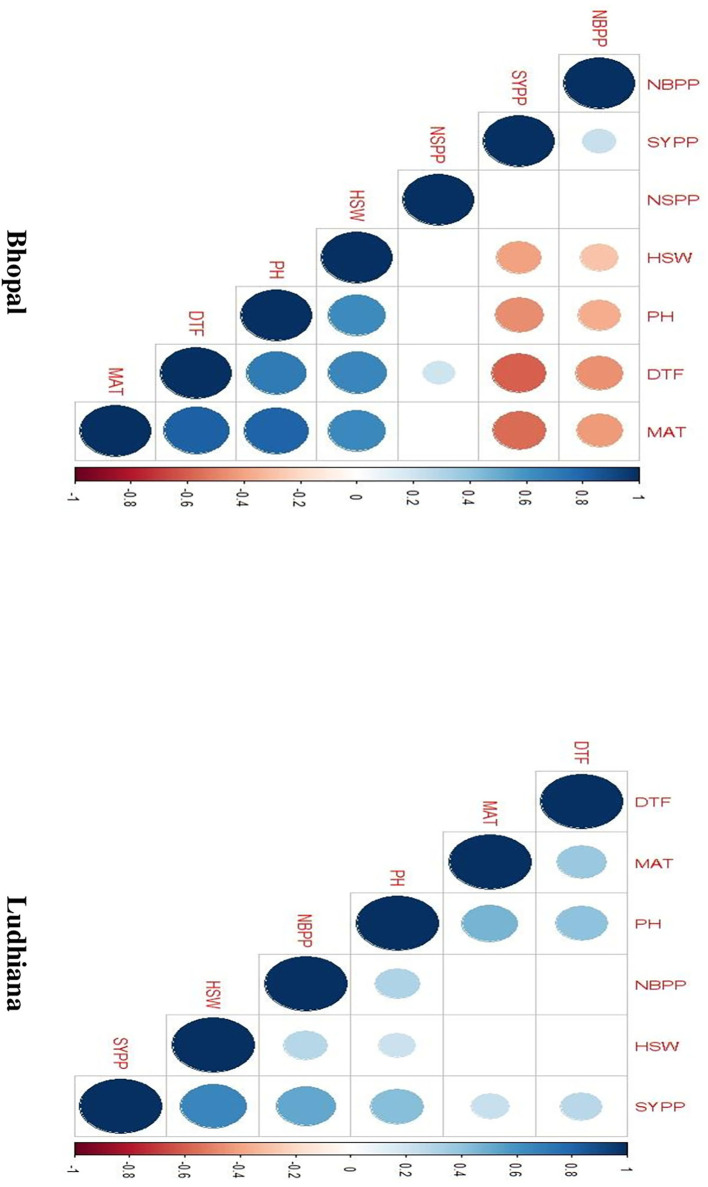
Correlation plots for agro-morphological traits among chickpea interspecific derivatives at Bhopal and Ludhiana, India. DTF, days to flowering; HSW, 100-seed weight; MAT, days to maturity, NBPP, number of branches plant^−1^; NSPP, number of seeds pod^−1^; PH, plant height; SYPP, seed yield plant^−1^.

**Figure 4:**
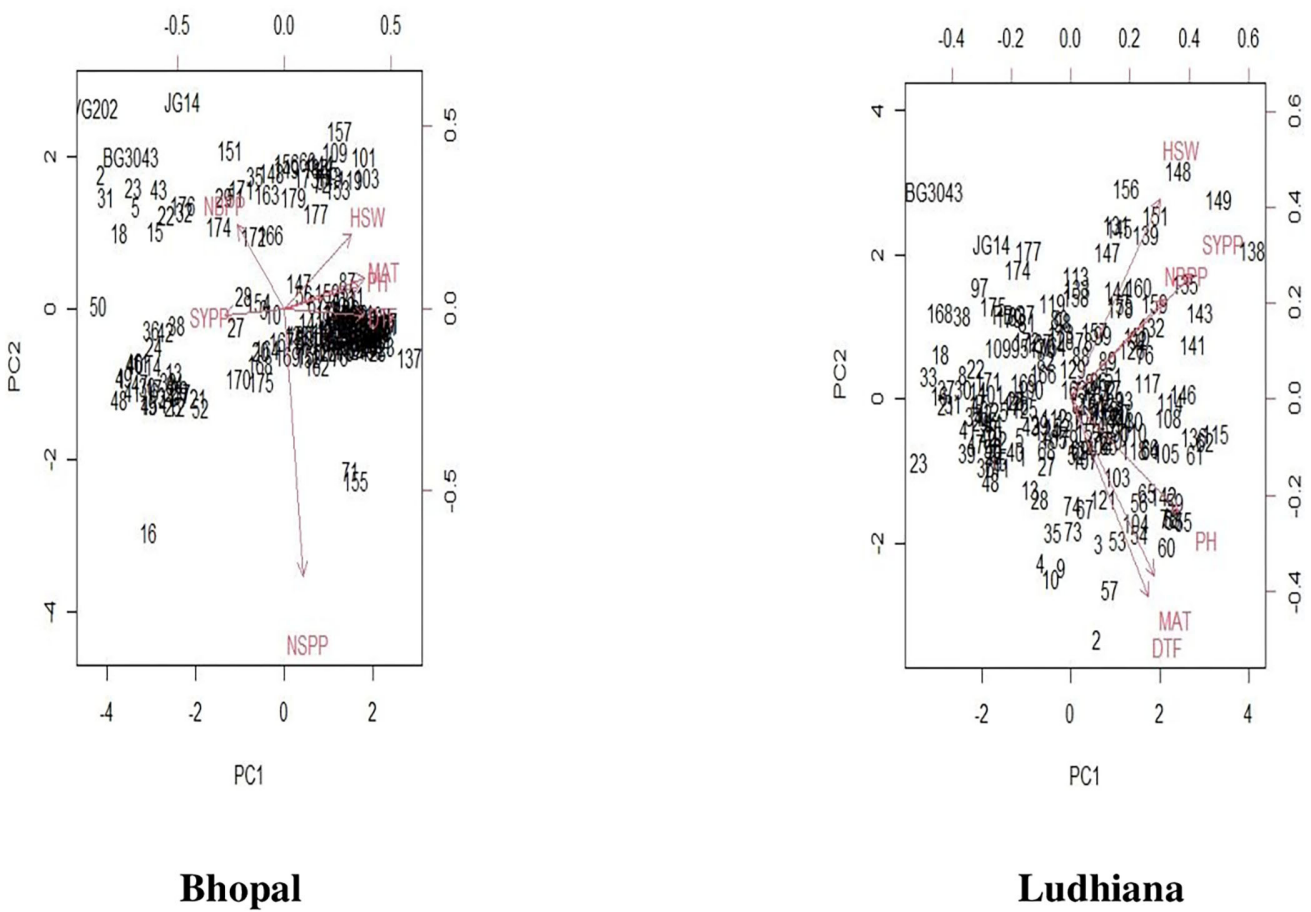
PCA biplots for chickpea interspecific derivatives at Bhopal and Ludhiana, India. DTF, days to flowering; HSW, 100-seed weight; MAT, days to maturity, NBPP, number of branches plant^−1^; NSPP, number of seeds pod^−1^; PH, plant height; SYPP, seed yield plant^−1^.

The association between days to flowering, days to maturity, and plant height showed positive correlations with each other for chickpea interspecific derivatives in Bhopal, India. Similarly, the number of branches plant^−1^ and seed yield plant^−1^ showed positive correlations in Ludhiana, India (Figure 3). Further, PCA biplots were used to ascertain this relationship among studied characters at both locations, but they gave contrasting results. It was observed that days to flowering, days to maturity, and plant height were highly correlated to each other while traits like the number of branches plant^−1^ and seed yield plant^−1^ showed negative correlations in Bhopal, India. However, in Ludhiana, India, days to flowering, days to maturity, and plant height exhibited negative correlations against the number of branches plant^−1^ and seed yield plant^−1^ which showed positive relations (Figure 4).

### Screening against major biotic stresses

#### Ascochyta blight

**Figure 5:**
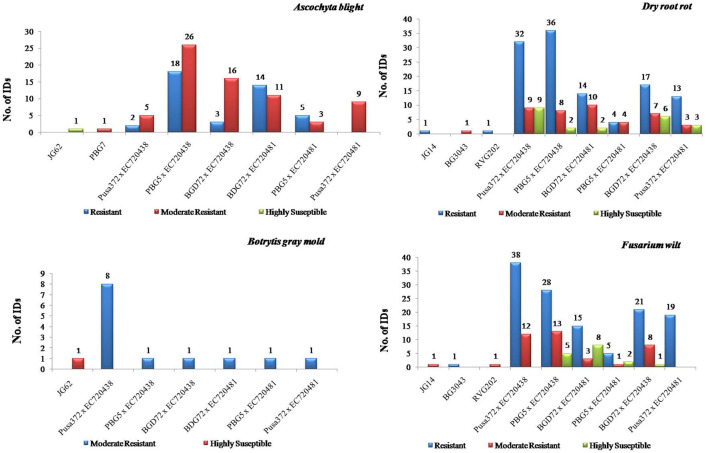
Performance of chickpea interspecific derivatives among different crosses and checks for ascochyta blight, botrytis gray mold, dry root rot, and fusarium wilt, respectively.

The selected agronomically promising F_7_ interspecific derivatives of Pusa372 × EC720438, PBG5 × EC720438, BGD72 × EC720438, BGD72 × EC720481, PBG5 × EC720481, and Pusa372 × EC720481 were screened against ascochyta blight resistance (Figure 5). The following interspecific derivatives of cross Pusa372 × EC720438 (2 IDs), PBG 5 × EC720438 (18 IDs), BGD72 × EC720438 (3 IDs), BGD 72 × EC720481 (14 IDs), and PBG5 × EC720481 (5 IDs) showed resistant disease reaction against the pathogen. Likewise, cross Pusa372 × EC720438 (5 IDs), PBG5 × EC720438 (26 IDs), BGD72 × EC720438 (16 IDs), BGD72 × EC720481 (11 IDs), PBG5 × EC720481 (3 IDs), and Pusa372 × EC720481 (9 IDs) were reported as moderately resistant against ascochyta blight (Figure 5). The remaining interspecific derivatives exhibited either susceptible or highly susceptible disease reaction. Further, the mean disease incidence score ranged in 1 to 9 scales with an overall mean of 6.18 and a coefficient of variation of 29.49%. However, susceptible (JG62) and resistant (PBG7) checks revealed disease reaction with a rating of 9 and 3 scores, respectively. The overall latent semantic indexing percentage (LSI %) against the disease infestation was 5.02 %.

#### Botrytis gray mold

The results of the screening of chickpea interspecific derivatives against botrytis gray mold revealed that none of the interspecific derivatives showed resistant disease reaction in real field conditions. But a moderate level of resistance has been reported in the following interspecific derivatives of Pusa372 × EC720438 (8 IDs), one each of PBG5 × EC720438, BGD72 × EC720438, BGD72 × EC720481, PBG5 × EC720481, and Pusa372 × EC720481. The overall latent semantic indexing percentage (LSI %) against the pathogen infestation was 7.02 % (Figure 5).

#### Dry root rot

The results of the screening of chickpea interspecific derivatives against dry root rot showed resistant disease reaction in the crosses of Pusa372 × EC720438 (32 IDs), PBG5 × EC720438 (36 IDs), BGD72 × EC720481 (14 IDs), PBG5 × EC720481 (4 IDs), BGD72 × EC720438 (17 IDs), and Pusa372 × EC720481 (13 IDs). Likewise, maximum moderate resistance was reported in the cross of Pusa372 x EC720438, followed by BGD72 × EC720481, PBG5 × EC720438, BGD72 × EC720438, and Pusa372 × EC720481. The remaining interspecific derivatives were either susceptible or highly susceptible to the disease reaction (Figure 5).

#### Fusarium wilt

The results of chickpea interspecific derivatives against fusarium wilt revealed that the crosses of Pusa372 × EC720438 (38 IDs), PBG5 × EC720438 (28 IDs), BGD72 × EC720481 (15 IDs), BGD72 × EC720438 (21 IDs), and Pusa372 × EC720481 (19 IDs) exhibited resistant disease reaction against the pathogen. Likewise, a moderate level of resistance has also been reported in the crosses of Pusa372 × EC720438 (12 IDs), PBG5 × EC720438 (13 IDs), BGD72 × EC720481 (3 IDs), and BGD72 × EC720438 (8 IDs). The remaining derivatives belonging to different crosses were either susceptible or highly susceptible to disease reaction (Figure 5).

## Discussion

Crop improvement programs are increasingly relying on pre-breeding and genetic enhancement employing CWRs to identify novel genes and alleles to broaden the genetic base of released cultivars (Singh et al., 2015). In chickpea, 41% of the crop varieties developed through hybridization had Pb7 as one of the ancestors in their pedigree (Kumar et al., 2003). The genetic base revealed by the pedigree records of released varieties appears to be narrow in major pulse crops, including chickpea, due to the frequent use of the same parents and their derivatives in breeding programs. To overcome these constraints, an attempt was, therefore, undertaken using wide hybridization of cultivated varieties (Pusa372, PBG5, and BGD72) taken as female and ILWC229 (EC720438) of *C. reticulatum* and ILWC246 (EC720481) of *C. echinospermum* taken as the male parents. True to type hybridity of F_1_ crosses was tested using Inter Simple Sequence Repeats (ISSR) and Random Amplified Polymorphic DNA (RAPD) markers (Singh et al., 2015). The experimental results revealed sufficient variation among genetic materials as indicated by mixed model analysis and analysis of variance (ANOVA) for the target traits (significant at *p* = 0.01, 0.05) assessed, and subsequently, the same was also reflected by range, mean, and coefficient of variation for important agro-morphological characters. However, descriptive statistics like skewness and kurtosis exhibited the normal distribution of expression performance for characters like the number of branches plant^−1^ and seed yield plant^−1^. Further, the interspecific derivatives provide a better opportunity for selecting promising transgressive segregants carrying potential traits of interest (Lewontin and Birch, 1966) along with classical genetic studies that have given a very compelling approach to the hypotheses that transgression can result from the expression of rare recessive alleles (Rick and Smith, 1953) and/or due to complementary gene action (Vega and Frey, 1980). Our results also exhibited the presence of transgressive segregations derived from *C. reticulatum* and *C. echinospermum*. All six interspecific derivatives were also assessed for their agronomic performance at two locations, including prevailing biotic stresses viz. ascochyta blight, botrytis gray mold, dry root rot, and fusarium wilt resistance under real field conditions. The genetic materials matured earlier in Bhopal, India than Ludhiana, India, suggesting certain physical factors and role of genotypic × environmental interactions, which could be responsible for the same. Erskine (1997) also reported the independent role of temperature and day length in determining the onset of ontogenesis in lentil. There were substantial variations with respect to plant height, the number of branches, seed number, and seed yield suggesting a good opportunity for selecting desirable ideotypes carrying important trait of interest (Eker et al., 2022). Further, hybrid vigor has also opened an era of genetic amelioration of crop plants and is often described as heterosis breeding. The fruitful heterosis also showed a wide range of variations among all interspecific derivatives for days to maturity, plant height, number of seeds pod^−1^, and seed yield plant^−1^. Similar results were also obtained by Singh and Ocampo (1997), Singh et al. (2005), and Singh et al. (2015) for yield-related traits in chickpea. An expression of the heterotic potential for certain interesting characters in advanced interspecific derivatives might be primarily due to the accumulation of favorable additive gene effects. Such derivatives may be advanced as suggested by Redden and Jensen (1974) for developing suitable genotypes. It was further indicated using estimation of other genetic parameters which revealed that high heritability and low genetic advance were predominantly assessed for the majority of characters indicating that non-additive gene effect and selection would be useful in later segregating generations when non-additive gene effect would have diminished. The genetic materials included in the study are interspecific cross populations belonging to different backgrounds carrying buffer hereditary information that lead to more transgression. Therefore, the estimate of heritability acts as a predictive approach in exercising reliability of phenotypic value, helping breeders to make a selection for a particular trait of interest when the heritability is high. Likewise, genetic advance is a useful indicator of progress, which can be expected as a result of exercising selection on population. Heritability in conjunction with genetic advance is more useful than heritability alone in predicting effects for selecting the best individual genotype because additive gene effects are likely to be present (Singh et al., 2014).

Furthermore, screening of chickpea enhanced progenies against major biotic stresses, which revealed that large numbers of interspecific derivatives were reported as resistant against ascochyta blight, indicating a substantial source of interspecific genetic resistance against the pathogen. Likewise, we were unable to find complete resistance against botrytis gray mold, but moderate resistance has been reported in 13 interspecific derivatives belonging to six cross-combinations of chickpea. However, for dry root rot and fusarium wilt, the interspecific derivatives exhibited resistance in various cross combinations of chickpea suggesting the potential of genetic materials to be taken further for developing disease-resistant cultivars. Overall, the wild *Cicer* species are a potential resource of useful untapped variations, including agro-morphological characters and major biotic stresses, as demonstrated by our findings. In F_7_ interspecific derivatives of chickpea, we found a wide range of variability for agro-morphological characters and major biotic traits, including considerable fruitful heterosis. It was also observed that derivatives derived from wild species showed more stability and yield levels, including resistance against major stresses. Thus, more emphasis should be given to the base-broadening program for tailoring usable germplasm with wider adaptations in future chickpea improvement programs. The potential lines carrying target traits could be a valuable genetic material for generation advancement to develop suitable genotypes. Lastly, useful genetic materials are being advanced for further breeding and desirable selection.

## Data availability statement

The original contributions presented in the study are included in the article/Supplementary material, further inquiries can be directed to the corresponding author.

## Author contributions

MS conceptualized the study and prepared the original draft. TK, UR, IS, and SB recorded data and performed field experimentation. SSo, NM, SSi, and SanjK helped in data analysis and manuscript editing. SandK helped in final editing of the manuscript. All authors contributed to the article and approved the submitted version.

## Conflict of interest

The authors declare that the research was conducted in the absence of any commercial or financial relationships that could be construed as a potential conflict of interest.

## Publisher's note

All claims expressed in this article are solely those of the authors and do not necessarily represent those of their affiliated organizations, or those of the publisher, the editors and the reviewers. Any product that may be evaluated in this article, or claim that may be made by its manufacturer, is not guaranteed or endorsed by the publisher.

## Abbreviations

CWRs: crop wild relatives
IDs: interspecific derivatives
ANOVA: analysis of variance
ILWC: international legume wild *Cicer*
EC: exotic collection
ICAR: Indian Council of Agricultural Research
NBPGR: National Bureau of Plant Genetic Resources
MAREC: Mountain Agriculture Research and Extension Center
ICARDA: International Center for Agricultural Research in Dry Areas
PAU: Punjab Agricultural University
BP: better parent
PBG: Punjab gram
BGD: Bangalore gram
PCA: principal component analysis
PCV: phenotypic coefficient of variation
GCV: genotypic coefficient of variation
SSD: single seed descent
ABD: augmented block design
FAO: Food and Agriculture Organization.
