# Supplementary material for: Identification of promising chickpea interspecific derivatives for agro-morphological and major biotic traits
Source: Front Plant Sci. 2022 Aug 4;13:941372. doi: 10.3389/fpls.2022.941372 (PMC9386514; doi:10.3389/fpls.2022.941372)
Supplement: Supplementary file 1 [file Data_Sheet_1.doc]

**Supplementary table 1: Mixed model combined analysis**

| Trait | Role | Genotypic variance | *p* value |
| --- | --- | --- | --- |
| DF | Gen | 11.38 | 0 |
| DF | Check | 0 | 1 |
| MAT | Gen | 0 | 1 |
| MAT | Check | 4.29 | 0.69 |
| PH | Gen | 15.38 | 0 |
| PH | Check | 24.26 | 4e-04 |
| NBPP | Gen | 4.99 | 0 |
| NBPP | Check | 7.74 | 0.20 |
| NSPP | Gen | 0.06 | 1 |
| NSPP | Check | 0.08 | 1 |
| HSW | Gen | 5.08 | 0 |
| HSW | Check | 6.41 | 0.01 |
| SYPP | Gen | 2.27 | 0 |
| SYPP | Check | 2.59 | 0.05 |
